# Molecular Imaging for Lung Cancer: Exploring Small Molecules, Peptides, and Beyond in Radiolabeled Diagnostics

**DOI:** 10.3390/pharmaceutics16030404

**Published:** 2024-03-15

**Authors:** Meliha Ekinci, Tais Monteiro Magne, Luciana Magalhães Rebelo Alencar, Pierre Basilio Almeida Fechine, Ralph Santos-Oliveira, Derya Ilem-Özdemir

**Affiliations:** 1 Department of Radiopharmacy, Faculty of Pharmacy, Ege University, 35040 Izmir, Turkey; meliha.ekinci@ege.edu.tr (M.E.); derya.ilem@ege.edu.tr (D.I.-Ö.); 2 Laboratory of Synthesis of Novel Radiopharmaceuticals and Nanoradiopharmacy, Nuclear Engineering Institute, Brazilian Nuclear Energy Commission, Rio de Janeiro 21941-906, Brazil; taismmagne@gmail.com; 3 Laboratory of Nanoradiopharmaceuticals and Radiopharmacy, State University of Rio de Janeiro, Rio de Janeiro 23070-200, Brazil; 4 Laboratory of Biophysics and Nanosystems, Department of Physics, Federal University of Maranhão, Campus Bacanga, Maranhão 65080-805, Brazil; luciana.alencar@ufma.br; 5 Grupo de Química de Materiais Avançados (GQMat), Departamento de Química Analítica e Físico-Química, Universidade Federal do Ceará—UFC, Campus do Pici, Fortaleza 60451-970, Brazil; fechine@ufc.br

**Keywords:** lung cancer, lung imaging, nuclear medicine, nanoradiopharmaceuticals, drug delivery system, drug targeting

## Abstract

It is evident that radiolabeled drug delivery systems hold great promise in the field of lung cancer management. The combination of therapeutic agents with radiotracers not only allows for precise localization within lung tumors but also enables real-time monitoring of drug distribution. This approach has the potential to enhance targeted therapy and improve patient outcomes. The integration of advanced imaging modalities, such as positron emission tomography (PET) and single-photon emission computed tomography (SPECT), has played a crucial role in the non-invasive tracking of radiolabeled drugs. These techniques provide valuable insights into drug pharmacokinetics, biodistribution, and tumor-targeting efficiency, offering clinicians the ability to personalize treatment regimens. The comprehensive analysis of preclinical and clinical studies presented in this review underscores the progress made in the field. The evidence suggests that radiolabeled drug delivery systems have the potential to revolutionize oncology by offering precise, targeted, and image-guided therapeutic interventions for lung cancer. This innovative approach not only enhances the effectiveness of treatment but also contributes to the development of personalized medicine strategies, tailoring interventions to the specific characteristics of each patient’s cancer. The ongoing research in this area holds promise for further advancements in lung cancer management, potentially leading to improved outcomes and quality of life for patients.

## 1. Introduction

Based on 2018 data provided by the International Agency for Research on Cancer (IARC), the global incidence of new cancer cases reached 18.1 million, resulting in 9.1 million cancer-related deaths [1]. Among men, lung cancer stands out as the most frequently diagnosed, accounting for 12–16% of cases, and it is also the most lethal, contributing to 18.4% of total cancer mortality. This high fatality rate is attributed to late-stage diagnoses and challenges in its treatment. Non-small-cell lung cancers (NSCLCs) make up approximately 85% of all lung cancers, while small-cell lung cancers (SCLCs) and neuroendocrine tumors constitute the remaining 15% [2]. Despite advancements in treatment, the overall survival rate for lung cancer patients beyond 5 years from diagnosis remains low, ranging from 10% to 15% [3]. Consequently, there is an imperative need for innovative approaches to enable the early diagnosis, treatment, and prevention of lung cancer.

Routine diagnostic procedures for lung cancer in clinical settings include chest radiography, computed tomography (CT), and nuclear medicine imaging methods such as positron emission tomography (PET) and single-photon emission computed tomography (SPECT). Additional diagnostic tools encompass sputum cytology, needle biopsy, bronchoscopy, and mediastinoscopy. Despite the availability of these non-invasive methods, they often fall short in detecting cancer in its early stages. Notably, nuclear medicine imaging methods are frequently favored due to their ability to generate three-dimensional images containing precise metabolic information, offering advantages over other diagnostic modalities [4].

In the diagnostic phase, chest radiography and CT scans are the preferred imaging modalities. For enhanced accuracy, additional procedures such as PET/CT, SPECT (single-photon emission computed tomography), and magnetic resonance imaging (MRI) can be employed.

[^18^F]F-FDG, a commonly used radiopharmaceutical in PET imaging studies, lacks specificity for lung cancer but is frequently utilized for metastasis detection, differentiation between benign and malignant lesions, and assessing treatment response [5]. While there are studies exploring radiopharmaceuticals for SPECT imaging, there is currently no specific radiopharmaceutical routinely used for imaging lung cancer in clinical practice [6].

Nanotechnology involves manipulating matter at the atomic and molecular levels to create new structures, altering their properties [7]. Nanomaterials in nanotechnology typically range from 1 to 100 nanometers (nm) in size [8]. Nanomedicine, a subset of nanotechnology, encompasses both diagnosis and therapy, utilizing the chemical, physical, electrical, optical, and biological properties of organic or synthesized nanoscale materials [7,9].

Nanoscale drug delivery systems offer advantages due to their biocompatibility, non-toxicity, and nonimmunogenicity [10,11,12]. These systems can be targeted to the tumor site, increasing bioavailability and allowing the transportation of multiple drugs or molecules to the tumor [13]. Active targeting strategies, achieved by reducing particle size or modifying the particle surface with hydrophilic polymers, as well as by conjugating passive and specific ligands to the particle surface, are effective methods employed with nanosized drug delivery systems [14,15,16,17].

Nanotechnology has emerged as a leading application in the field of medicine, particularly in cancer diagnosis and treatment, or theranostics [13]. Nanoparticles play a crucial role in overcoming biological barriers and mechanisms of drug resistance. They contribute to increased circulation time, enhanced bioavailability, and reduced toxicity. The synergy of “nanotechnology” and “nuclear medicine” facilitates the creation of innovative nanoparticulate drug carriers [18]. This review specifically concentrates on radiolabeled drug delivery systems designed for the diagnosis of lung cancer.

## 2. Drug Delivery Systems

Nanotechnology holds significant promise in the medical field, with potential applications encompassing the development of drug delivery systems and diagnostic devices [19]. Diverse drug delivery systems have emerged from the application of nanotechnology to pharmaceuticals, including nanoparticles, nanospheres, nanocapsules, liposomes, niosomes, emulsions, polymeric systems, dendrimers, colloid gold, quantum dots (QDs), micelles, sphingosomes, microbubbles, microspheres, and superparamagnetic particles [20].

Compared to conventional drugs, the reduction in particle size results in an increased superficial area, leading to higher surface area exposure. Additionally, nanosized drugs typically exhibit 100–500% higher accumulation than agents in the angstrom size range [21]. These nanosized drugs enhance safety and efficacy by increasing bioavailability while reducing side effects [22,23].

**Figure 1:**
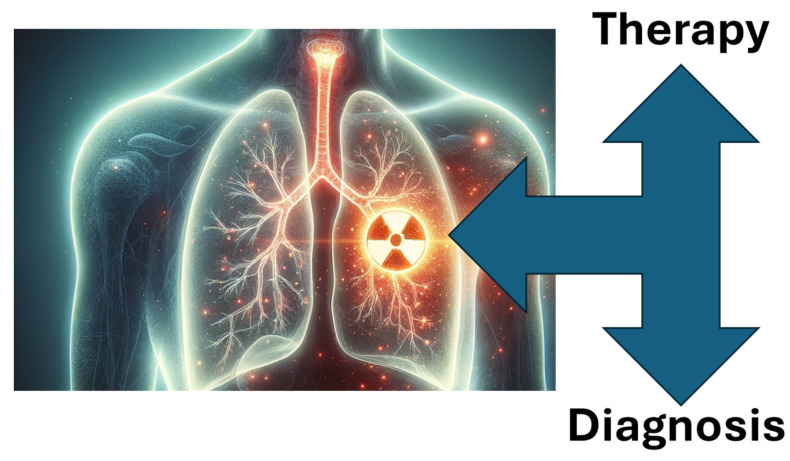
Application of radioactive nanoparticles for both imaging and therapy modalities.

Nanosystems are broadly categorized into therapeutics and imaging [24] (Figure 1). When nanosystems are employed for diagnostic purposes, they are expected to remain stable under physiological conditions without breaking down. Conversely, those used for treatment require disintegration [25,26].

The advantages of utilizing nanoparticular systems are outlined as follows [27,28]:

**Enhanced Circulation**: The small size of nanoparticles allows drugs to easily traverse veins and participate in the circulation of blood flow.

**Improved Imaging Quality**: Nanoparticles with nanometer-scale sizes contribute to increased surface areas, thereby enhancing the quality and resolution of images.

**Increased Solubility**: Nanoparticle size leads to improved solubility, resulting in enhanced absorption and bioavailability of the particles.

**Targeted Delivery:** Nanoparticles can be more precisely targeted to specific patients and desired areas, facilitating more effective and localized drug delivery.

**Suitability for Biological Treatments**: Nanopharmaceuticals are well suited for biological treatments, providing a platform for innovative therapeutic approaches.

**Multi-drug Loading**: Nanocarriers can accommodate more than one active substance, allowing for the loading of multiple therapeutic agents onto a single nanocarrier.

**Versatility in Targeting Molecules**: Multiple targeting molecules can be attached to nanocarriers, enabling a sophisticated level of precision in directing therapeutic interventions.

These advantages underscore the potential of nanoparticular systems in advancing drug delivery, imaging, and therapeutic strategies in the field of medicine.

## 3. Targeting of Drug Delivery Systems

Targeting in drug delivery aims to selectively increase the concentration of the drug in a specific region compared to other areas by directing the active substance to the intended action or absorption site. The primary objectives of drug targeting include optimizing the interaction by releasing the drug precisely at the site of action and at the desired rate. This approach also seeks to reduce the dose of the active substance and constrain its distribution solely to the target organ. Drug delivery systems achieve targeting in two distinct ways [29].

### 3.1. Passive Targeting

Passive targeting involves delivering drugs to specific sites primarily through natural physiological processes and factors. This approach capitalizes on the inherent anatomical disparities between normal and pathological tissues to effectively transport drugs to the intended site [30]. Research has demonstrated that, in certain cases, such as tumor cells, there is an increase in the permeability of blood vessel walls. The loose vascularization of tumors allows the drug delivery system to spontaneously penetrate the interstitium from the blood vessel walls. This phenomenon is referred to as the enhanced permeability and retention effect (EPR effect) [31].

### 3.2. Active Targeting

Active targeting involves specific interactions between the drug delivery system and target cells, often characterized by ligand–receptor interactions [30]. At its core, active targeting relies on the utilization of targeted ligands capable of binding specifically to receptor structures directed toward the target, such as antibodies and peptides. This can be achieved through various methods, including antibodies, peptide conjugation, pH, O_2_, or heat-sensitive carriers [32]. The success of active targeting hinges on the careful selection of targeting tools with high affinity for cell surface receptors, coupled with appropriate chemical modifications for forming effective conjugations. It may involve ligand–receptor or antigen–antibody interactions, as well as the use of targeting aptamers that identify pathological cells with distinct molecules concentrated at the site of pathology. The targeted therapeutic agent aims for a high accumulation of drugs in the pathological structure with the assistance of a carrier that can bind with cell- or tissue-specific ligands. Consequently, nanosystems of varying sizes, in addition to their ability to combine with different targeting ligands, offer promising opportunities for overcoming physiological barriers and facilitating efficient cellular uptake of drugs. Various nanosystems can achieve higher concentrations than conventional drugs in cellular uptake [33,34].

Diseases often exhibit a significant increase in endothelial cell surface receptors. Targeting carriers to these receptors not only aids in imaging but also facilitates treatment [35]. In malignant tumors, angiogenesis is essential for tumor viability and growth. The widely expressed α_v_β_3_ integrin in the vascular endothelium of tumors, due to angiogenesis, can be specifically reached by peptide-coated carriers. Experimental studies have demonstrated that systemically injected nanoparticle–α_v_β_3_ integrin complexes successfully target the tumor, leading to tumor shrinkage through apoptosis of the vascular endothelium [36].

## 4. Drug Delivery Systems in Nuclear Medicine for Lungs

**Figure 2:**
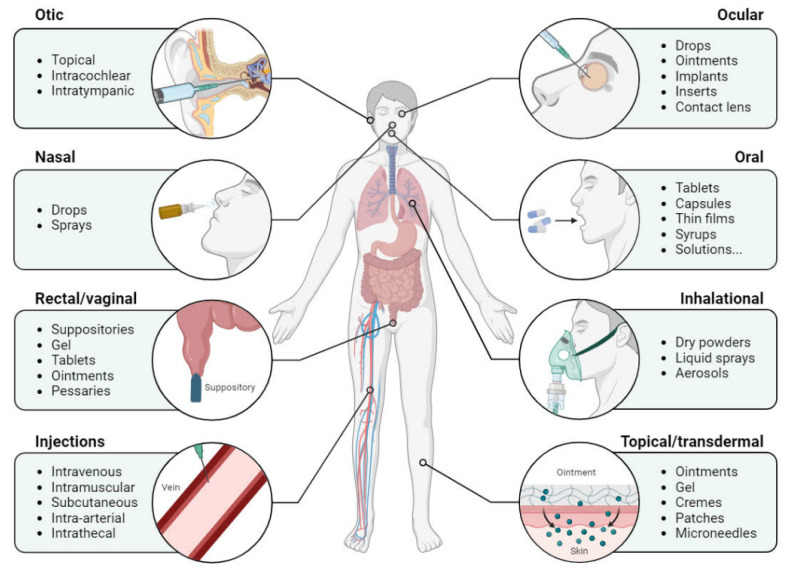
Main routes for drug administration.

In recent years, there has been a significant emphasis on drug release for lung applications due to its potential in treating a diverse array of both local and systemic diseases [37,38,39]. Pulmonary delivery has garnered interest not only for the local treatment of airway diseases but also for the systemic administration of drugs (Figure 2). The lung presents several advantages as an application route, including its expansive surface area, thin alveolar epithelium, easily permeable membrane, and large vasculature. These characteristics enable the rapid and high absorption of soluble and permeable actives [40,41].

Various inorganic nanoparticles, including micelles, microbubbles, dendrimers, liposomes, and other polymeric structures, can be labeled with suitable radionuclides and employed in both diagnosis and therapy through passive and active targeting strategies [42]. SPECT and PET modalities offer advantages such as non-invasiveness, high sensitivity, and the ability to use labeled molecules for multiple imaging sessions and quantitative analysis, including dynamic processes. However, these modalities have the disadvantage of lower resolution, which can be addressed by enhancing intrinsic and extrinsic resolution and improving accuracy through multimodal imaging approaches [43].

While some preclinical and clinical studies have been conducted in the literature on radiopharmaceuticals intended for SPECT imaging, there is currently no specific radiopharmaceutical established for lung cancer imaging in clinical practice [6]. Numerous preclinical and phase studies have explored the use of radiopharmaceuticals labeled with [^111^In]In, [^201^Tl]Tl, and [^99m^Tc]Tc for clinical imaging of lung cancer. Details of radiolabeled nanoparticles in various lung cancer models are provided in Table 1.

A study conducted by Spanu et al. involving 111 patients evaluated the effectiveness of [^99m^Tc]Tc-tetrofosmine in distinguishing between benign and malignant non-calcified solitary pulmonary nodules (SPNs) smaller than 3 cm. The SPECT images demonstrated that the [^99m^Tc]Tc-tetrofosmine radiopharmaceutical exhibited a sensitivity of 91.7%, a specificity of 88.9%, and an accuracy of 91% in characterizing these lesions [44].

In a multi-center phase 3 study, SPECT images taken with [^99m^Tc]Tc-depreotide, a somatostatin analog, aimed at differentiating benign and malignant SPN lesions smaller than 6 cm. [^99m^Tc]Tc-depreotide scintigraphy was found to be safer, more cost-effective, and more useful than PET imaging with [^18^F]FDG, displaying high sensitivity (96.6%), specificity (73.1%), and accuracy (91%) [6]. Subsequent studies with [^99m^Tc]Tc-depreotide for lung cancer also yielded similar results [45,46].

In a retrospective study, the efficiency of SPECT/CT images taken using [^99m^Tc]Tc-octreotide, another somatostatin analog, in distinguishing between benign pulmonary nodules and cancerous tissues was investigated. The specificity rates for diagnosing pulmonary malignant nodules were 63.6%, 72.7%, and 81.8% for images obtained with diagnostic CT, SPECT/CT, and the combination of diagnostic CT and SPECT/CT, respectively. These findings suggest that the combination of diagnostic CT and SPECT/CT is more specific than diagnostic CT alone in characterizing malignant lesions [47].

**Table 1:** Representative studies evaluating radiolabeled particles in vitro, in vivo, and in clinical models of lung cancer.

Radionuclide	Nanoparticles/Chelate Agent	Mean Particle Diameter/Experimental Conditions	Radiochemical Purity (%)	Cell Line, Animal, or Clinical Model	Clinical Prospects and Applications	Ref.
[^99m^Tc]Tc	Tetrofosmin	-	>95%	Solitary pulmonary nodule patients	[^99m^Tc]Tc-tetrofosmin SPECT as highly sensitive imaging method in both primary and secondary malignant ≤ 3 cm SPNs	[44]
[^99m^Tc]Tc	Etoposide microparticles/chelate-free	430 ± 10.2 nm; 10 min/25 °C	>99%	A549 cells and male Balb/c nude mice	Biodistribution and SPECT imaging for early diagnosing of lung cancer	[48]
[^99m^Tc]Tc	PLA/PVA/Atezolizumab nanoparticles	-	>99%	L-929 and A-549; mice	Accurate imaging for NSCLC detection	[49]
[^99m^Tc]Tc	Zolmitriptan	45 min/25 °C	92.5%	Male Swiss albino mice	Potential radiopharmaceutical for lung scintigraphy safer than what is commercially available	[50]
[^99m^Tc]Tc	Liposome/DTPA	150–180 nm; 30 min/25 °C	>80%	A549 and H1299 cells	Theranostic nanosized, radiolabeled, co-drug-encapsulated liposomes’ potential for the diagnosis and therapy of NSCLC	[51]
[^99m^Tc]Tc	Gold nanoparticles (AuNPs)	30 min/80 °C	99%	4T1-luc-GFP cell line; female BALB/c mice	Advantages of vascular targeting and nanotechnology to effectively target breast cancer metastasis	[52]
[^99m^Tc]Tc	Stealth liposomal doxorubicin (Caelyx)/DTPA	~100 nm	-	Preclinical models of NSCLC and HNC	Treatment of locally advanced NSCLC	[53]
[^99m^Tc]Tc	Solid lipid nanoparticles/HMPAO	10 min/25 °C; 200 nm	97 ± 2%	Adult male Wistar rats	Biodistribution, lymphoscintigraphy, and therapy upon pulmonary delivery	[54]
[^188^Re]Re	Liposome/BMEDA	84.6 ± 4.12 nm; 30 min/60 °C	71.1%	NCI-H292 cells	Chemotherapy and radiotherapy using NCI-H292 cells	[55]
[^188^Re]Re	Human serum albumin microspheres/chelate-free	1 h/95 °C/pH 2; 25 μm	>90%	Wistar rats	Biodistribution profiles in Wistar rats for lung radiotherapy	[56]
[^131^I]I	Immuno-gold-nanoparticles/chelate-free	52.9 nm	>95%	A549 tumor-bearing mice.	MicroSPECT/CT images of A549 tumor-bearing mice	[57]
[^124^I]I	LHRH-modified human serum albumin-stabilized gold nanoclusters	6 ± 0.5 nm; 10 min/25 °C	>98%		NIR fluorescence and PET imaging agent for early diagnosis in lung cancer model	[58]
[^125^I]I	Polyacrylamide nanoparticles/chelate-free	20–40 nm; 30 min/25 °C	>95%	Human bronchial epithelial cell line (BEAS 2B)	Biodistribution and microPET imaging using BEAS 2B for lungs	[59]
[^111^In]In	Perfluorocarbon nanoparticles	242 nm	85 to 90+%	White rabbit	α_v_β_3_-integrin-targeted [^111^In]In nanoparticles for clinical use to identify occult tumors or metastases and guide follow-on high-resolution MRI.	[60]
[^18^F]F	Icotinib	100 min/50 and 80 °C	>99%	A549 xenograft mice	PET imaging of A549 xenograft mice	[61]
[^18^F]F	MPG	10 min/120 °C and 35 °C	>99%	NSCLC patients	PET for non-invasive imaging and quantification of EGFR-activating mutation status in preclinical models of NSCLC	[62]
[^177^Lu]Lu	Chitosan nanoparticles/chelate-free	30 min/25 °C/pH 5; 165 ± 10 nm	98.6 ±1.2%	Epithelial lung cancer cells and C57BL/6 mice	Radionuclide therapy. In vitro results using epithelial lung cancer cell lines	[63]
[^177^Lu]Lu	Cellulose nanocrystals/DOTA	60 min/100 °C/pH 4; 136–158 nm	74 ± 2%	BRAF V600E mutant cell lines (murine YUMM1.G1 and human A375) and wild-type BRAF cell line (murine B16-F10)	Biodistribution, chemotherapy and radionuclide therapy. In vitro and in vivo results using a lung metastatic melanoma model	[64]
[^64^Cu]Cu	Polyglucose nanoparticles (Macrin)/NODAGA	30 min/90 °C/pH 6; ~20 nm	>99%	MC38 cells in C57BL/6 immunocompetent mice and MC38 xenograft model	PET and optical imaging of tumor-associated macrophages in lung carcinoma	[65]

**[^18^F]F-MPG:** N-(3-chloro-4-fluorophenyl)-7-(2-(2-(2-(2-18F-fluoroethoxy) ethoxy) ethoxy) ethoxy)-6-methoxyquinazolin-4-amine; **BMEDA:** N,N-bis(2-mercaptoethyl)-N′,N′-diethylethylenediamine; **CT:** computed tomography; **DOTA:** 1,4,7,10-tetraazacyclododecane-1,4,7,10-tetraacetic acid; **DTPA:** diethylenetriaminepentaacetic acid; **EGFR:** epidermal growth factor receptor; **HMPAO:** hexamethylpropyleneamine oxime; **LHRH:** luteinizing hormone-releasing hormone; **MRI:** magnetic resonance imaging; **NIR:** near-infrared; **NODAGA:** 1,4,7-triazacyclononane, 1-glutaric acid-4,7-diacetic acid; **NSCLC:** non-small-cell lung cancer; **PLA:** poly lactic acid; **PVA:** poly vinyl alcohol; **SPECT:** single-photon emission computed tomography.

In a study comparing [^99m^Tc]Tc-MIBI and [^201^Tl]Tl-chloride, the response of NSCLC patients to chemotherapy was evaluated based on the accumulation rates in tumor tissue [66]. The [^99m^Tc]Tc-MIBI group exhibited significantly higher rates of delayed tumor involvement uptake in contralateral normal lung tissue and uptake index in the responding group compared to the non-responding group. Conversely, there was no correlation between groups in the images taken with [^201^Tl]Tl-chloride. This suggests that the [^99m^Tc]Tc-MIBI radiopharmaceutical may be more effective than [^201^Tl]Tl-chloride in assessing chemotherapy response in NSCLC patients [66].

In another study, etoposide microparticles were prepared and radiolabeled with [^99m^Tc]Tc as a diagnostic agent for early-stage lung cancer imaging [48]. The results indicated that over 10% of the total dose used was taken up by the tumor site. Additionally, the microparticles exhibited good renal clearance and low uptake by the reticuloendothelial system (RES). Researchers concluded that the developed micro-radiopharmaceuticals could be utilized for SPECT imaging of lung cancer [48].

Polymeric nanoparticles composed of PLA/PVA/Atezolizumab were radiolabeled with [^99m^Tc]Tc for the early diagnosis of NSCLC [49]. These nanoparticles demonstrated high labeling efficiency (≥99%) using a thin-layer chromatography (TLC) system. The results showed no cytotoxic effect on normal cells (L-929) but exhibited cytotoxicity on tumor cells (A-549). Biodistribution assays demonstrated that [^99m^Tc]Tc-PLA/PVA/Atezolizumab could reach the tumor site more than 14 times after 1 h compared to non-particulate Atezolizumab, indicating its potential as a new drug for obtaining accurate images of lung cancer [49].

In a study by Rashed et al. (2016), zolmitriptan (a selective serotonin receptor agonist) was radiolabeled with [^99m^Tc]Tc and assessed for its potential application in lung imaging. Research on biodistribution revealed that 15 min following administration, the peak pulmonary absorption of [99mTc]Tc-zolmitriptan reached approximately 23.89 ± 1.2% of the dose administered per gram of tissue, with significant retention observed in the lungs for up to an hour. The primary pathway for clearance was identified as renal. Scintigraphic imaging corroborated these findings, displaying high-quality images of the lungs with minimal radioactivity observed in other organs, barring the kidneys and bladder. The safety profile of [99mTc]Tc-zolmitriptan was deemed superior compared to the existing [99mTc]Tc-MAA, and its pulmonary uptake was greater than that of newly discovered agents like [^123^I]I-PMPD, [^99m^Tc]-Tc(CO)5I, and [^99m^Tc]Tc-DHPM. Consequently, [^99m^Tc]Tc-zolmitriptan emerged as a promising radiopharmaceutical for lung scintigraphy imaging [50].

Xu et al. designed theranostic gold nanoparticles for use in lung cancer [67]. [^198^Au]Au nanoparticles were prepared for cancer therapy, while [^99m^Tc]Tc-Au nanoparticles were prepared for cancer imaging. Both gold nanoparticles demonstrated significant efficiency for both the therapy and diagnosis of lung cancer, as evaluated by the MTT methodology using A549 cells and in vivo tumor imaging for [^99m^Tc]Tc-Au nanoparticles [67].

Lin et al. [55] investigated the theranostic efficacy of a [^188^Re]Re-conjugated liposomal formulation coated with polyethylene glycol (PEG) in lung cancer, comparing it with a [^188^Re]Re-N,N-bis(2-mercaptoethyl) N′.N′diethylethylenediamine (BMEDA) conjugate. Biodistribution and imaging studies using nano-SPECT/CT in a xenograft tumor model revealed that liposomal [^188^Re]Re was detected in the tumor area for up to 48 h, while [^188^Re]Re-BMEDA rapidly moved away from the tumor site. Additionally, liposomal [^188^Re]Re exhibited a longer stay in the blood, slower clearance, and a higher maximum concentration level reached in the blood compared to [^188^Re]Re-BMEDA conjugate. The results indicated that the liposomal formulation demonstrated these properties and tumor-targeting ability in vivo, attributed to its EPR effect. Based on these findings, it was reported that the liposomal [^188^Re]Re formulation is a promising theranostic agent suitable for SPECT imaging in lung cancer [55].

Kao et al. [57] synthesized iodine-131 [^131^I]I-radiolabeled gold nanoparticles [^131^I]I-C225-AuNPs-PEG for targeted SPECT/CT imaging of the epidermal growth factor receptor (EGF-R) using cetuximab, with the aim of achieving a therapeutic effect. To assess the impact of cetuximab on nanoparticle cellular uptake, confocal laser microscope images from A549 cells treated with [^131^I]I-C225-AuNPs-PEG in the presence and absence of C225 were compared. The results showed a decrease in fluorescent signals and cellular uptake of nanoparticles in the presence of C225, indicating the specific binding of C225-AuNPs-PEG to EGF-R. MTT results demonstrated the biocompatibility of non-radioactive nanoparticle formulations (C225-AuNPs-PEG and AuNPs-PEG). In a biodistribution study conducted 2 and 4 h after injection, the ratio of radioactivity in the tumor to muscle radioactivity was calculated as 3.9 and 5.5, respectively, suggesting that nanoparticles were taken up by the tumor tissue within 4 h in the experimental animal. The functionalized, [^131^I]I-radiolabeled, gold nanoparticles were identified as potential theranostic agents suitable for SPECT imaging [57].

Clinical applications of labeling macroaggregate albumin (MAA) and human serum albumin (HSA) microspheres with [^68^Ga]Ga, as drug delivery systems, have commenced after preclinical studies. The first clinical study, conducted by Kotzarka et al. [68] in 2010, involved lung ventilation with [^68^Ga]Ga-labeled carbon particles (Galligas) and lung perfusion PET/CT with [^68^Ga]Ga-HSA microspheres in 15 patients with suspected pulmonary embolism. The method was reported to be particularly successful in demonstrating lung perfusion [68].

Han et al. [58] developed iodine-124 [^124^I]I-labeled gold nanoclusters as a PET imaging agent for lung tumors. Modified Au nanoclusters were synthesized by conjugating luteinizing hormone-releasing hormone (LHRH) with HSA as a scaffold. Tumor-targeting characteristics were evaluated using A549 and orthotopic xenograft tumor models. Orthotopic lung tumors could be clearly visualized for 2 to 5 h after the injection of [^124^I]I-LHRH-HSA AuNCs. Fluorescence imaging also confirmed tumor-targeting properties, highlighting potential applications for the early diagnosis of lung cancer.

Polyacrylamide-based hydrogel micro- and nanoparticles functionalized with the cell penetration peptide nona-arginine (Arg9) were radiolabeled with [^76^Br]Br and [^125^I]I by Liu et al. [59] for PET imaging and biodistribution studies, respectively. Flow cytometry evaluation using Alexa Fluor 488 dye-conjugated particles in a human bronchial epithelial cell line revealed significantly higher intracellular uptake of Arg9-modified nanoparticles compared to unmodified ones. Intratracheal injection of [^76^Br]Br-labeled nanoparticles in C57BL/6J mice demonstrated prolonged lung retention for nanosized particles compared to microsized ones in PET images. Furthermore, SUV comparisons indicated that microparticles modified with Arg9 exhibited lower lung retention than unmodified ones, while the retention rates for modified and unmodified nanoscale particles were similar for 18 h. Biodistribution study results showed that most activity in all particles localized in the lungs, with nanoscale particles remaining in the lungs longer than microsized particles. Notably, nanoscale Arg9-modified particles exhibited significantly higher uptake compared to non-modified ones. The study concluded that this nanoscale system holds promise for optical and nuclear imaging of lung cancer, suggesting the need for in vivo testing via routes other than intratracheal administration [59].

The epidermal growth factor receptor (EGFR) has gained interest as a target for imaging in NSCLC. Lu et al. [61] radiolabeled icotinib, an EGFR-tyrosine kinase inhibitor (EGFR-TKI), with [^18^F]F to develop a targeted PET probe, EGFR-[^18^F]F-icotinib. PET images of mice bearing A549 EGFR-positive xenografts demonstrated specific uptake of [^18^F]F-icotinib (0.90 ± 0.24% ID/g) with high contrast between the tumor and normal tissue. Rapid probe clearance from non-target organs, such as the lung, muscle, stomach, kidney, and liver within 90 min post-injection, resulted in low background uptake and high tumor-to-normal organ contrast in the images. Co-injection of icotinib with [^18^F]F-icotinib significantly reduced [^18^F]F-icotinib uptake in tumors, indicating the specific binding of this EGFR-mediated probe.

Sun et al. [62] developed a new PET imaging agent, [^18^F]F-MPG, for determining epidermal growth factor receptor (EGFR) mutation status in the treatment of NSCLC. The study concluded that [^18^F]F-MPG PET/CT is a powerful method for definitively determining EGFR-activating mutation status in NSCLC patients. This non-invasive approach enables the identification of patients susceptible to EGFR tyrosine kinase inhibitors (TKIs) and monitoring the effectiveness of EGFR TKI therapy, providing a promising strategy for patient management [62].

Combined therapy is defined as using more than one drug or method in the treatment of a disease. In a study, nanosized, folate receptor-targeted, co-drug-encapsulated (paclitaxel and vinorelbine), radiolabeled liposomal formulations were designed for the effective diagnosis and treatment of NSCLC [51]. The uptake of these actively targeted theranostic liposomes was higher in H-1299 cells (NSCLC cell line) than in passively targeted formulations, and the actively targeted formulations showed a higher uptake in H-1299 cells than in A-549 cells (lung cancer cell line). Empty liposomal formulations were found to exhibit a biocompatible profile on cells, and the anti-cancer efficacy of co-drug-encapsulated liposomes was found to be significantly higher than that of a single-drug-encapsulated liposomal formulation. Based on these results, the researchers highlighted the potential of theranostic nanosized, radiolabeled, and adjuvant-encapsulated liposomes for the diagnosis and treatment of NSCLC [51].

## 5. Lung Cancer Targeting Probes

The development of probes has consistently been a central focus of molecular imaging, aiming to achieve accurate disease diagnosis at an early stage and assess therapeutic responses. Numerous strategies have been employed to create new optical imaging probes [69,70,71]. An ideal clinical probe should exhibit a high target-to-background ratio to maximize in vivo image contrast. Additionally, it should possess characteristics such as high binding affinity, target-specific uptake and retention, rapid clearance from non-target tissues, stability and integrity in vivo, ease of preparation, and safe clinical use [69,72].

In contrast to optical fluorophores, various radionuclides can be conjugated to affinity ligands for the development of new radiolabeled probes. The half-life of the isotope used should be long enough to complete synthesis and administration to the patient but not longer than necessary [73]. Numerous scintigraphy probes have been presented previously, and some have found successful applications in clinical research. The growing understanding of overexpressed peptide-binding receptors in specific tumors has spurred interest in developing peptide-based probes through radiolabeling techniques [74,75,76].

Peptide probes are typically designed to act as regulators, mimicking natural peptides that vary in size from a few to tens of amino acids. These peptides play a crucial role in modulating physiological conditions through specific receptors and high-affinity peptide binding [74].

Xin Zhou et al. (2022) evaluated for the first time in humans the biodistribution, metabolism, radiation dosimetry, safety, and potential of [^68^Ga]Ga-NOTA-WL12 as a PET probe in quantifying programmed death ligand-1 (PD-L1) expression levels in patients with advanced NSCLC. [^68^Ga]Ga-NOTA-WL12 PET imaging proved to be safe with acceptable radiation dosimetry. Physiological tracer uptake was visible mainly in the liver, spleen, small intestine, and kidney. Tumors were clearly visible, particularly in the lungs, with a tumor-to-lung ratio of 4.45 ± 1.89 at 1 h. One hour was a suitable time for image acquisition, as no significant differences were observed in tumor-to-background ratios between 1 and 2 h. A strong, positive correlation was found between tumor uptake (SUV_peak_) and immunohistochemistry for PD-L (r = 0.9349; *p* = 0.002) [77]. The quantification of PD-L1 by a radiolabeled probe was also evaluated by Marc et al. (2020). The uptake of [^18^F]F-BMS-986192, an adnectin PET tracer of PD-L1 in patients with NSCLC, was evaluated in the study. However, additional validations were recommended [78].

In the last decade, several peptides containing RGD, sequences that target the extracellular domain of integrin receptors, have been radiolabeled and evaluated [79,80,81]. The integrin family regulates critical cellular functions in the progression of solid tumors and, consequently, has made them an attractive target for theranostic application [82,83,84]. The α_v_β_3_ integrin receptors are highly expressed in proliferating growing tumor cells of different origins, including NSCLC. The ability of a [^68^Ga]Ga-radiolabeled DOTA-E peptide (cRGDfK)_2_ to act as a PET tumor imaging agent in non-small-cell lung carcinoma was investigated [85].

The radiolabeled peptide’s cell uptake study shows high uptake by A549 cells, indicative of αvβ3-positive integrin presence. [^68^Ga]Ga-DOTA-E(cRGDfK)_2_ exhibited a targeted affinity for α_v_β_3_ receptors, highlighting its specificity in binding. Roughly 16.5% of [^68^Ga]Ga-DOTA-E(cRGDfK)2 was internalized by the cells, with 83.5% adhering to the cell surface following a 2 h incubation period. The rate of nonspecific binding was around 35%. In vivo studies on A549 tumors revealed markedly elevated tumor accumulation and minimal residual radioactivity in other organs, aside from noticeable radioactivity levels observed in the kidneys and intestines. PET/CT imaging using [^68^Ga]Ga-DOTA-E(cRGDfK)_2_ demonstrated significantly high tumor uptake [85].

The α_v_β_6_ integrin has been identified as a significant marker for lung cancer, typically linked to a worse prognosis. Ren et al. [86] created the cystine-knot peptide R01-MG, intended for PET imaging of α_v_β_6_-positive lung cancer cases. R01-MG was crafted through solid-phase peptide synthesis techniques and labeled with [^68^Ga]Ga following its conjugation with DOTA, a cyclic compound designed for radiolabeling. In vivo PET scans and distribution studies showed that [^68^Ga]Ga-DOTA-R01-MG was characterized by its quick and efficient uptake in α_v_β_6-_expressing lung tumors, along with swift elimination from non-cancerous tissues, which enhanced the contrast between tumor and normal tissue in images. Conversely, the absence of significant uptake of [^68^Ga]Ga-DOTA-R01-MG in α_v_β_6_-negative lung cancer models underscored the probe’s targeted specificity towards the α_v_β_6_ integrin [86].

Flechsig et al. (2019) investigated the clinical value of SFITGv6, a specific α_v_β_6_ integrin peptide, for the targeted diagnosis of NSCLC. PET/CT scans conducted on patients diagnosed with NSCLC after administering [^18^F]FDG, followed by the application of [^68^Ga]Ga-SFITGv6, demonstrated aligned imaging results. When comparing the uptake levels of [^68^Ga]Ga-SFITGv6 to those of [^18^F]FDG, both sets of PET/CT images revealed a marked elevation in SUVmax values within lesions confirmed to be NSCLC through histology. Nonetheless, it was generally observed that [18F]FDG exhibited a higher degree of accumulation [87]. Conjugating radionuclides with antibodies represents an innovative method for creating specialized scintigraphic imaging agents. The progastrin-releasing peptide (ProGRP), known for its oncogenic influence in SCLC, is targeted by the monoclonal antibody anti-ProGRP(31-98) D-D3, which has shown selective accumulation in SCLC xenografts within mice. In a study by Hong et al. (2020), the effectiveness of a pre-targeted biotin–avidin system (BAS) in conjunction with anti-ProGRP(31-98) D-D3 was explored for SCLC diagnosis, focusing on assessing biodistribution and radioimmunoimaging (RII) efficacy in mice with SCLC xenografts. D-D3 was labeled with [^99^mTc]Tc through a tri-step pre-segmentation technique, and this pre-segmentation approach was evaluated against direct radiolabeling methods regarding biodistribution and RII outcomes. The pre-targeting strategy, as opposed to direct labeling of D-D3, demonstrated a specific improvement and signal intensification in the tumors, potentially enhancing the uptake of [^99^mTc]Tc-D-D3 by the targeted tumors and offering a novel method for the early detection of SCLC [88]. Radiolabeled recognition probe systems in different lung cancer models are shown in Table 2.

## 6. Drug Delivery Systems and Recognition in Angiogenesis and Lung Metastasis

Vascular endothelial growth factor receptor (VEGF-R) and α_v_β_3_ integrin play important roles in tumor growth and angiogenesis, and they are targeted for cancer diagnosis and treatment [95]. SPECT imaging of lung cancer angiogenesis using α_v_β_3_ integrin-targeted [^111^In]In-labeled perfluorocarbon nanoparticles has been performed, and it has been suggested that they may be ideal diagnostic agents in newly formed tumors [60].

In a recent investigation, Wang et al. [89] performed a pilot cohort study to assess the viability of [^68^Ga]Ga-FAPi-RGD, a bifunctional PET tracer targeting both fibroblast-activating protein (FAP) and α_v_β_3_ integrin, in identifying lung cancers. This tracer was evaluated alongside [^18^F]F-FDG and the single-function tracers [^68^Ga]Ga-RGD and [^68^Ga]Ga-FAPi for lung cancer detection efficacy. The findings endorsed [^68^Ga]Ga-FAPi-RGD PET/CT as a superior diagnostic tool for lung cancer. Specifically, [^68^Ga]Ga-FAPi-RGD PET/CT outperformed [^18^F]F-FDG in primary tumor detection rate (91.4% vs. 77.1%, *p* < 0.05), exhibited higher tumor uptake (SUV_max_, 6.9 ± 5.3 vs. 5.3 ± 5.4, *p* < 0.001), and demonstrated a greater tumor-to-background ratio (10.0 ± 8.4 vs. 9.0 ± 9.1, *p* < 0.05). Additionally, it achieved superior precision in evaluating mediastinal lymph nodes (99.7% vs. 90.9%, *p* < 0.001) and detected more metastatic sites (254 vs. 220). A notable distinction was also observed in the uptake of primary lesions between [^68^Ga]Ga-FAPi-RGD and [^68^Ga]Ga-RGD (SUVmax, 5.8 ± 4.4 vs. 2.3 ± 1.3, *p* < 0.001) [89]. Radiolabeled fibroblast-activating protein (FAPI) inhibitors are significant markers for the non-invasive imaging of tumor microenvironments. They enable the evaluation of FAP expression in fibroblasts, macrophages, and cells associated with cancer, providing a crucial tool for understanding the involvement of these elements in the tumor landscape. FAPI is often used as a protumorigenic stromal marker [96]. The diversity observed within the tumor microenvironment is largely attributed to the coevolution and persistent interaction between stromal and tumor cells throughout the process of tumorigenesis [97]. This dynamic interplay is a key contributor to the complexity seen in tumor biology [98]. FAPI’s ability to specifically target PAF enables its utilization in PET/CT imaging, facilitating the identification of PAF within the tumor setting. This capability provides critical insights into the biological traits of tumors, enhancing our understanding of their behavior and potential vulnerabilities.

Wei et al. (2022) showed that the [18F]F-AlF-NOTA-FAPI-04 PET/CT scan demonstrates the capability to differentiate between various lung cancer types by assessing the tracer uptake in primary and metastatic lesions. It has uncovered variability in PAF expression across lung cancer metastases, notably showing the most significant expression in bone metastases. Consequently, the [18F]F-AlF-NOTA-FAPI-04 PET/CT scan could play a crucial role in devising treatment strategies for individuals with advanced stages of lung cancer, offering targeted insights for personalized therapy planning [90].

The primary cause of mortality in breast cancer patients is attributed to the metastatic spread of the disease [99]. Although the delivery of nanoparticles to deep tissue is effective for certain primary tumors, targeting the vascular system presents a more appealing approach for addressing micrometastases [100,101,102,103]. These smaller, widespread metastatic formations pose a significant challenge for molecular or nanoscale therapeutic agents due to biological hurdles such as their diminutive size and extensive dispersion to various organs, making them nearly impervious to conventional treatments [104,105,106,107,108]. To overcome these obstacles in reaching micrometastatic sites, Peiris et al. [52] have developed an innovative method that integrates vascular targeting techniques with the advanced targeting properties of gold nanoparticles, offering a new avenue for effectively addressing micrometastatic breast cancer.

The αvβ3 integrin plays an important role in the development of micrometastases at a distal site and was used as a vascular targeting strategy. For the purpose of tracking the distribution of nanoparticles at metastatic locations, gold nanoparticles (AuNPs) were tagged with [^99^mTc]Tc. Studies conducted on animals and analyzed through histology demonstrated that the vascular-directed delivery of these nanoparticles enabled precise targeting of micrometastases in the 4T1 mouse model of breast cancer metastasis. This was achieved through radionuclide imaging techniques and employing a minimal dosage of nanoparticles. The strategic approach to targeting resulted in the deposition of 14% of the administered AuNPs into metastatic sites within the lungs merely an hour after injection. This finding underscores the potential of the metastatic vascular bed as an effective target for nanoparticle-based interventions [52].

[^18^F]F-AlF-NOTA-PRGD2 ([^18^F]F-Alfatide), a new tracer targeting α_v_β_3_ integrin, has been studied for PET imaging of angiogenesis [109,110]. Zhou et al. (2017) conducted a pilot investigation of the diagnostic ability of [^18^F]F-Alfatide PET/CT in imaging detection of mediastinal lymph node metastases in patients with NSCLC.

In a study examining lymph node (LN) metastases, 20 out of 196 dissected LNs were identified as malignant through pathological examination. All malignant LNs were effectively detected using [^18^F]F-Alfatide PET/CT scans in the patients studied. The metrics of SUV_max_, SUV_mean_, and SUV ratios for malignant LNs showcased significantly higher values compared to those in benign LNs among patients with non-small-cell lung cancer (NSCLC), with statistical significance (*p* < 0.001). These findings were consistent across patients diagnosed with both adenocarcinoma and squamous cell carcinoma (*p* < 0.001). The [^18^F]F-Alfatide PET/CT displayed high diagnostic accuracy with sensitivity ranging from 83.9% to 100%, specificity between 78.6% and 96.7%, and precision from 81.7% to 96.9%, based on thresholds derived from the receiver operating characteristic curve. These outcomes indicate the significant utility of [^18^F]F-Alfatide PET/CT in identifying metastatic LNs in individuals with NSCLC [91].

Recently, Guo et al. (2022) investigated the role of the RGD-dimerized [^18^F]F-Alfatide PET marker ([^18^F]F-Alfatide-RGD) in NSCLC. Forty-eight patients with NSCLC were included in the study. The [^18^F]F-Alfatide-RGD PET/CT imaging demonstrated reliable accuracy in identifying primary lung cancer sites and showed high consistency in the detection of patients with positive and metastatic lymph nodes, aligning well with pathological findings. In the initial diagnosis phase of NSCLC, [^18^F]F-Alfatide-RGD exhibited notable sensitivity and specificity, significantly aiding in the precise clinical staging and preoperative assessment. Further examination of the advantages of [^18^F]F-RGD PET/CT highlighted its capability to reveal increased angiogenesis within the NSCLC tumor microenvironment, particularly notable in cases of squamous cell carcinoma. This underscores the value of [^18^F]F-RGD PET/CT not only in diagnostic accuracy but also in enhancing the understanding of the pathological characteristics of NSCLC, contributing to more informed clinical decision-making [93].

Melanoma, recognized for its aggressive malignancy, has a propensity for early metastasis to the lungs, making the non-invasive tracking of metastatic melanoma crucial for tailoring appropriate treatment protocols. In response to this need, Tanaka et al. introduced the development of radiolabeled γ-PGA ternary complexes, specifically [^111^In]In-DTPA-G4/[^125^I]I-PEI/γ-PGA, designed for the nuclear medical imaging of lung metastases from melanoma. Their research revealed that both forms of the radiolabeled γ-PGA complexes were absorbed by melanoma cells in a manner dependent on the duration of exposure. Notably, there was a significantly higher cellular uptake of the [^125^I]I component compared to the [^111^In]In within the complexes [^111^In]In-DTPA-G4/[^125^I]I-PEI and [^111^In]In-DTPA-G4/[^125^I]I-PEI/γ-PGA, highlighting the potential effectiveness of these probes in the imaging and detection of metastatic melanoma in the lungs.

On the other hand, [^125^I]I radioactivity was eliminated from the blood faster than [^111^In]In. In an in vivo experiment employing the [^111^In]In-DTPA-G4/[^125^I]I-PEI/γ-PGA probe, it was found that the liver, spleen, and lungs exhibited nearly identical levels of radioactivity from both [^125^I]I and [^111^In]In shortly after the administration of the probe. Notably, in the lungs, the retention of [^125^I]I was extended in comparison to [^111^In]In. Further, a biodistribution study conducted on mice with melanoma metastasized to the lungs revealed that there was a relatively higher accumulation and a three-fold increase in the lung-to-blood ratio of [^111^In]In-DTPA-G4/[^125^I]I-PEI/γ-PGA in the lungs affected by metastatic melanoma compared to the lungs of healthy mice. This suggests that the γ-PGA complex is effectively targeted and taken up by B16-F10 melanoma cells, demonstrating its potential as a probe for the specific imaging of metastatic melanoma in the lung [94].

Sobic-Saranovic et al. [111] compared [^99m^Tc]Tc labeled HMFG1 monoclonal antibody ([^99m^Tc]Tc-HMFG1), [^201^Tl]Tl-chloride, and [^99m^Tc]Tc-Sestamibi in terms of their sensitivity in NSCLC and evaluated their power to distinguish tumor tissue by detecting tumor and distant metastases. In light of the results obtained, it was seen that the sensitivities of [^201^Tl]Tl-chloride and [^99m^Tc]Tc-Sestamibi were significantly higher than the sensitivity of [^99m^Tc]Tc-HMFG1, and it was concluded that these two radiopharmaceuticals could be considered as potential agents in terms of taking whole-body images in detecting distant metastases in NSCLC patients [111].

The development of radiolabeled nanomaterials as tracers in medical diagnostics and therapy presents a multidisciplinary challenge that intersects the fields of nanotechnology, radiopharmacy, and oncology. These challenges are fundamentally rooted in the complex interplay between the physical characteristics of nanomaterials and the radiophysical properties of isotopes, particularly their half-lives, alongside the translational potential for clinical application. This discussion will explore these intricacies, the current state of development, and the prospective future directions of radiolabeled nanomaterial probes.

The integration of radionuclides with nanomaterials to create effective tracers for diagnostic and therapeutic purposes is a sophisticated process influenced by the half-life of isotopes. The half-life of a radionuclide—the time required for half of the radionuclide to decay—poses a significant challenge in the design and synthesis of radiolabeled nanomaterials. Short-lived isotopes, while advantageous for reducing radiation exposure to the patient, demand rapid synthesis, labeling, and application before significant decay occurs. This necessitates the development of highly efficient and rapid synthesis and labeling techniques, which can be technically demanding and cost-intensive. Conversely, long-lived isotopes, though offering a wider window for application and potentially broader distribution and usage, raise concerns about prolonged radiation exposure and the consequent risk of radiotoxicity. Balancing these considerations requires a nuanced understanding of the intended clinical application, including the kinetics of nanomaterial accumulation and clearance in target versus non-target tissues.

The potential clinical applications of radiolabeled nanomaterials are vast and include targeted drug delivery, image-guided therapy, and personalized medicine. The unique properties of nanomaterials—such as high surface area, the ability to tailor surface properties for specific targeting, and the capacity to encapsulate or bind to various drugs and imaging agents—make them highly versatile platforms for radiopharmaceutical development.

One of the critical areas of focus has been the development of targeted radiolabeled nanoparticles for cancer diagnostics and therapy. These nanoparticles can be engineered to recognize and bind to specific biomarkers overexpressed on cancer cells, allowing for targeted imaging and therapy. This targeted approach aims to improve the specificity and sensitivity of cancer detection and treatment, minimizing damage to healthy tissues.

The future of radiolabeled nanomaterial probes lies in overcoming the current challenges through innovative research and development. Advances in nanotechnology, radiochemistry, and molecular biology are crucial for the next generation of nanomaterial tracers. Future directions may include the following: i) development of new radiolabeling techniques: innovative methods that are rapid, efficient, and capable of attaching radionuclides to nanoparticles without compromising the integrity and functionality of the nanomaterial; ii) hybrid nanosystems: combining different types of nanomaterials or modalities (e.g., magnetic nanoparticles with radiolabels) to leverage the advantages of each system for enhanced imaging and therapeutic efficacy; iii) biomarker discovery and targeting: identification of new biomarkers for various diseases and the development of targeting ligands or antibodies that can be used to guide the nanomaterials to diseased tissues with high specificity; iv) safety and biocompatibility assessment: rigorous evaluation of the long-term safety, biocompatibility, and potential toxicity of radiolabeled nanomaterials is essential for translating these technologies from bench to bedside.

In conclusion, the development of radiolabeled nanomaterials as tracers is a complex and evolving field that stands at the intersection of several advanced scientific disciplines. While the challenges are significant, particularly in balancing the physical and chemical properties of nanomaterials with the radiophysical properties of isotopes, the potential clinical applications offer considerable promise for the future of medical diagnostics and therapy. Continued interdisciplinary research and collaboration are vital to realizing the full potential of these innovative probes in the clinical setting.

## 7. Conclusions

Lung cancer is a prevalent malignancy with a poor prognosis. The stage has an inverse relationship with survival, and early detection and diagnosis are critical for a surgical cure. The use of multifunctional drug delivery systems in both diagnosis and treatment contributes significantly to personalized treatment, as it provides multimodal imaging guidance and treatment response monitoring. It is thought that with the increase in clinical studies in the future, these systems will have a much wider place in the diagnosis and treatment of lung cancer, which is the most common cause of death.

## Figures and Tables

**Table 2:** Representative studies evaluating radiolabeled recognition probe systems in vitro, in vivo, and in preclinical models of lung cancer.

Radionuclide	Recognition Probe Systems	Experimental Conditions	Radiochemical Purity (%)	Cell Line, Animal, or Clinical Model	Clinical Prospects and Applications	Ref.
[^68^Ga]Ga	NOTA-WL12	15 min/60 °C	>99%	Chinese hamster ovary (CHO); non-small-cell lung cancer patients	Complementary diagnostic to immunohistochemistry to quantify PD-L1 levels for patient selection and therapeutic monitoring in anti-PD-L1 therapy	[77]
[^68^Ga]Ga	DOTA-E(cRGDfK)_2_	5 min/125.4 °C	>%98	A549 cells; Swiss male mice	Radiotracer for cancer imaging, tumor treatment response monitoring, and follow-up PET imaging	[85]
[^68^Ga]Ga	DOTA-R_0_1-MG	-	-	A549, H1975 and H1299 lung cancer cell lines	Potential to be a PET tracer for imaging α_v_β_6_-positive lung cancer	[86]
[^68^Ga]Ga	FAPI-RGD	10 min/95 °C/pH 4.5	95%	Model lung neoplasm patients	PET [^68^Ga]Ga-FAPI-RGD increases tumor uptake and retention, tumor-targeting efficiency, and pharmacokinetics compared to monospecific markers	[89]
[^68^Ga]Ga [^125^I]I [^177^Lu]Lu	SFITGv6	-	-	NSCLC cell lines; NCI-H2009 and NCI-H322 tumor-bearing mice	Affinity and binding properties of SFITGv6 for α_v_β_6_ integrin-expressing NSCLC cell lines and SFITGv6-PET/CT scan to distinguish between malignant and inflammatory lesions	[87]
[^99m^Tc]Tc	Biotinylated D-D_3_; DTPA-biotin	30 min/30 °C; 15 min/25 °C; 4 h/4 °C.	92.14 ± 3.55%	NCI-H446 cell line; female athymic nude mice	Pre-targeting technology using D-D_3_ and [^99m^Tc]Tc signal amplification increases tumor radiopharmaceutical concentration and enables early diagnosis of small-cell lung cancer	[88]
[^18^F]F	BMS-986192	45 min/45 °C	>95%	Models of advanced-stage NSCLC patients eligible for nivolumab treatment	Quantification of PD-L1 expression with PET/CT in patients with advanced-stage non-small-cell lung cancer	[78]
[^18^F]F	AlF-NOTA-FAPI-04	10 min/110 °C	98%	Model lung cancer patients	[^18^F]F-AlF-NOTA-FAPI-04 PET/CT imaging for quantification of FAP in metastases of lung cancer, especially in bone metastases, of different pathological types of lung cancer	[90]
[^18^F]F	Alfatide	5–30 min/70–110 °C/pH 2–6	> 95%	NSCLC patients	[^18^F]F-Alfatide PET/CT imaging for diagnosing metastatic lymph nodes with a high sensitivity and specificity in patients with NSCLC	[91]
[^18^F]F	Alfatide	-	-	NSCLC patients	[^18^F]F-Alfatide-RGD PET/CT for diagnosis and pathologic results of primary foci, lymph-node-positive, and metastatic lymph nodes of NSCLC	[92]
[^18^F]F	RGD	5–30 min/70–110 °C/pH 2–6	> 95%	NSCLC patients	[^18^F]F-RGD PET/CT imaging for detection of angiogenesis in the tumor microenvironment of NSCLC	[93]
[^111^In]In [^125^I]I	DTPA-G4/PEI/γ-PGA	1 h/25 °C/pH 6 and 9	>90%	B16-F10 cell line; male BALB/c mice	γ-PGA complex for nuclear medical diagnosis of lung-metastatic melanoma and the possibility of use as multimodality imaging probes	[94]

**CT:** computed tomography; **DTPA:** diethylenetriaminepentaacetic acid; **FAP:** fibroblast activation protein; **FAPI:** fibroblast activation protein inhibitor; **NOTA:** 2,2′,2″-(1,4,7-triazacyclononane-1,4,7-triyl)triacetic acid; **NSCLC:** non-small-cell lung cancer; **PD-L1:** programmed death ligand-1; **PEI:** polyethyleneimine; **PET:** positron emission tomography; **PGA:** polyglutamic acid; **RGD:** Arg-Gly-Asp.

## Data Availability

All data will be available upon request.
